# Value of Transvaginal Two-Dimensional Contrast-Enhanced Ultrasonography in Diagnosing Atypical Ovarian Corpus Luteum Hematoma

**DOI:** 10.1155/2018/3120579

**Published:** 2018-10-09

**Authors:** Hui Liu, Hong Xiang, Ruixue Mu, Palida Tuoerhan, Qianqian Zeng, Huili Zhou, Rong Hu, Gulinaer Shahai

**Affiliations:** ^1^Department of Obstetrics and Gynecology Ultrasound, Ultrasound Medical Center, The First Affiliated Hospital of Xinjiang Medical University, Xinjiang 830054, China; ^2^Department of Obstetrics and Gynecology, Tacheng Hospital of Traditional Chinese Medicine, Xinjiang, 834700, China

## Abstract

**Objective:**

To evaluate the value of transvaginal two-dimensional contrast-enhanced ultrasonography (2D-CEUS) in the diagnosis of atypical ovarian corpus luteum hematoma (AT-OCLH).

**Methods:**

A prospective study was performed on 53 consecutive patients with suspected AT-OCLH, and the diagnostic results by transvaginal 2D-CEUS were statistically compared with the gold standard. The gold standard results were confirmed by surgical pathology or long-term follow-up.

**Results:**

The characteristic perfusion patterns of AT-OCLH in 2D-CEUS showed no contrast agent perfusion within the tumor mass, and the capsule wall showed rapid, annular, high enhancement perfusion; perfusion patterns could be classified into type Ia and type IIa. AT-OCLH can be distinguished from ovarian tumors based on perfusion characteristics and perfusion pattern type, which can be diagnosed based on the significantly stronger cystic wall perfusion intensity, earlier arrival time, and thicker cystic wall than nonluteal cystic foci (P<0.05). The sensitivity of 2D-CEUS for diagnosing AT-OCLH was 95.7%, with a specificity of 96.6%. A 2D-CEUS scoring system for AT-OCLH was established. Lesions with scores >17.5 were diagnosed as AT-OCLH.

**Conclusion:**

2D-CEUS is an effective method for diagnosing AT-OCLH.

## 1. Introduction

Although ovarian corpus luteum hematoma is often found in the female pelvic cavity by ultrasound examination, the different internal echo changes of the mass often make it difficult to diagnose [1]. The various changes in the sonographic features of ovarian corpus luteum hematoma are related to its formation [2, 3]. Large amounts of internal bleeding within the normal corpus luteum would lead to the formation of corpus luteum hematoma. Early luteal hematomas with relatively large amounts of intracystic bleeding manifest as near-round cysts within the ovary with thick and/or rough cystic walls and messy inhomogeneous low echo within the cyst, showing diversified echo pattern and sometimes nearly inhomogeneous solid echo. In midterm corpus luteum hematoma, blood coagulates within the cyst with partial absorption, along with a thinning of the capsule and smooth inner walls, reduced echo in the cyst, and fine reticular structure. For late-stage luteal hematoma, the cyst becomes smaller after blood absorption, showing slightly higher solid echo within the cyst, the boundary of which with surrounding ovarian tissue is not clear, forming a corpus luteum cyst after complete absorption of blood with smooth cyst walls and no internal echo. The color Doppler flow imaging (CDFI) of luteal hematoma often demonstrates the following characteristics: typical annular or semiannular blood flow signals around the luteal cyst wall.

When a corpus luteum hematoma becomes cystic (simple cystic or with fine reticular structure within the cyst), with CDFI showing a typical annular or semi-annular blood flow signal around the capsule wall, it is considered a typical ovarian corpus luteum hematoma (T-OCLH), which can be diagnosed by two-dimensional (2D) gray scale ultrasound, CDFI examination, and/or short-term follow-up.

When the luteal hematoma appears mixed (presents mixed echo/messy and inhomogeneous hypo-echogenicity within the cyst) or near inhomogeneous solid echo, showing solid high echogenicity at the late stage of luteal hematoma formation with an unclear boundary with the surrounding ovarian tissue, and the nourishing vessels near the ovarian medulla are difficult to identify, it is defined as atypical ovarian corpus luteum hematoma (AT-OCLH).

It is difficult to diagnose AT-OCLH with conventional 2D gray scale and CDFI techniques [4], as it is difficult to differentiate from ovarian solid or mixed tumors, chocolate cysts, or mature cystic teratomas, of which the treatments are completely different in clinical practice. As AT-OCLH is physiological masses that usually disappear after a few months of regular follow-up, they generally do not need to be treated except for acute abdominal surgery in cases of hematoma rupture [5, 6]. Ovarian tumors [7, 8], chocolate cysts, and mature cystic teratomas require surgery, medicine, and other active treatments [9, 10]. This study aimed to find a better diagnostic method for AT-OCLH, which is significant for clinicians to make correct treatment plans, avoid unnecessary surgeries, and reduce the burden on hospitals and patients.

The current diagnostic methods for corpus luteum hematoma reported in the literature are still mostly transvaginal 2D ultrasound and CDFI technologies [11], which achieve a satisfactory diagnosis of T-OCLH, but have a high misdiagnosis rate for AT-OCLH.

Contrast-enhanced ultrasound is a new method for imaging tissue vascular perfusion [12, 13]. Through peripheral intravenous injection of microbubble contrast agents and using a harmonic and pulse inversion technique of contrast-enhanced ultrasound and effective contrast-enhanced ultrasound imaging and Doppler flow signal of parenchymatous organs, it overcomes the effect of depth and size of the mass on the effectiveness of imaging blood flow rate, which is a supplement to 2D ultrasound and color Doppler imaging [14, 15]. There are no reports on contrast-enhanced ultrasound diagnosis for AT-OCLH. This study utilized transvaginal 2D-CEUS on mass inspection of 53 suspected cases of AT-OCLH, with prospective diagnostic analysis study design, to explore the diagnostic accuracy of 2D-CEUS in AT-OCLH.

## 2. Materials and Methods

### 2.1. Study Subjects

#### 2.1.1. Inclusion Criteria

(1) Inclusion criteria were consecutive patients who had not entered menopause and (2) solid and mixed echogenic foci suspected of atypical ovarian corpus luteum by 2D gray scale and CDFI examination.

#### 2.1.2. Exclusion Criteria

(1) Exclusion criteria were postmenopausal patients; (2) typical ovarian luteal hematoma (simple cystic or fine mesh structure within the cyst, with typical annular or semiannular blood flow around the cystic wall shown by CDFI); (3) typical fibroma (significant posterior echo attenuation); (4) patients allergic to contrast agent, or with serious heart, lung, or kidney dysfunction, pregnant and lactating women, minors under the age of 18, and patients with serious drug allergy.

A total of 53 patients admitted in the Department of Obstetrics and Gynecology ultrasound, at the First Affiliated Hospital of Xinjiang Medical University from August 2009 to December 2015 who met the inclusion criteria, were enrolled in the study. The age range was 19–50 years, with a mean age of 36.4 ± 7.8 years. All patients were examined before signing the consent agreement. This research project was approved by the ethics committee of First Affiliated Hospital of Xinjiang Medical University in March 2009 (approval number: 20090324).

### 2.2. Instruments and Methods

#### 2.2.1. Instruments and Reagents

This study utilized the MyLab90 color Doppler ultrasound diagnostic instrument with Contrast Tuned Imaging technique (Esaote, Genova, Italy). We also utilized a transvaginal probe frequency of 5–9 MHz, with a low mechanical index of 0.08 focused on the bottom level of the lesion. The ultrasound contrast agent SonoVue (Bracco, Milan, Italy) was used.

#### 2.2.2. Inspection Process

Inspection process included the following: (1) transvaginal 2D ultrasound and CDFI were done to record lesion location, size, boundary, internal echo, blood flow distribution, and arterial resistance index spectrum of the lesions and surrounding areas; (2) to determine the region of interest (ROI) by contrast-enhanced examination, we selected the solid part of the lesion and the area with the most abundant blood supply shown by CDFI as ROI; (3) transvaginal 2D-CEUS examination: we selected the ROI after conversion to 2D-CEUS imaging mode, keeping the probe position unchanged. At the same time, we dissolved 59 mg of lyophilized contrast agent in 5 mL saline solution. After mixing, 2.4 mL of the microbubble suspension was injected through patients' cubital veins with a syringe flushed with 5 mL saline. Upon injection, the ultrasound timer and workstation dynamic acquisition function were opened, with continuous and dynamic observation of the contrast agent perfusion and subsidence time in the lesion and surrounding tissue, as well as the contrast agent perfusion within the lesions, until complete subsidence of the contrast agent. The imaging process was no less than 5 min with dynamic imaging recorded. (4) Analysis of the contrast-enhanced image was done. After image collection, the following data were obtained: (i) 2D-CEUS perfusion pattern of the adnexal mass (Table 1); (ii) grading of the perfusion intensity by 2D-CEUS (compared with the perfusion intensity of surrounding tissue) (Table 2); and (iii) extracting the dynamic image storage for quantitative analysis using Qontrast 4.0 software (QONTRAST-QRADIO, Esaote Spa, Italy) for offline analysis, with the ROI determined as the region of the highest perfusion intensity within the lesion. The distribution of the contrast enhancement effect in each ROI over time was reflected by the time-intensity curve (TIC), following: Peak Intensity (PI), Time to Peak (TTP), Mean Transit Time (MTT), and Arrival Time (AT). (5) Analysis of 2D-CEUS images of masses was performed by two experienced physicians with certificates for contrast-enhanced ultrasound. The gold standard results were confirmed by surgical pathological examination or long-term follow-up. All patients underwent surgical treatment within 1 week after contrast-enhanced ultrasound, ensuring synchronization between the results of contrast-enhanced ultrasound and surgical pathological examination. After the initial scanning, patients were examined regularly at 1, 2, 3, and 6 months until the masses disappeared; patients for whom the masses did not disappear underwent surgical treatment with pathological confirmation. Before operation, these patients were re-examined by 2D-CEUS to ensure synchronization between the contrast-enhanced ultrasound and surgical pathological results.

#### 2.2.3. Clinical Trial Registration

This study was registered in the China Clinical Trial Registration Platform on April 2011 (http://www.ctrni.org), and the registration number is ChiCTR-DNC-11001305.

#### 2.2.4. Statistical Analysis

Data were analyzed using SPSS version 16.0 statistical software package (SPSS Inc., Chicago, IL, USA). Measurement data did not meet the normal distribution, and were represented as M (*P*25,* P*75). Count data were analyzed using the rank sum test, with* P*<0.05 considered statistically significant. The sensitivity, specificity, positive predictive value, negative predictive value, positive likelihood ratio, negative likelihood ratio, and coincidence rate of 2D-CEUS in the diagnosis of AT-OCLH were calculated.

#### 2.2.5. Funding

This project was supported by the National Natural Science Foundation of China (project number: 30960101) and the Natural Science Foundation of Xinjiang autonomous region (project number: 200821144). These organizations were not involved in the study design, data collection, data analysis, or research reports.

## 3. Results

**Figure 1:**
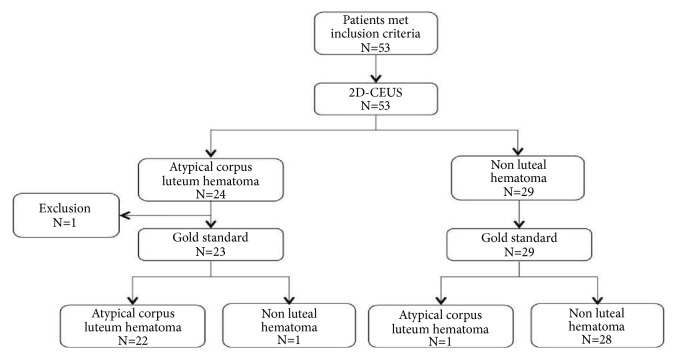
Patient enrollment flowchart.

*3.1.* Patient enrollment information is summarized in Figure 1. The adnexal mass of 53 patients met the inclusion criteria, and these patients were subjected to 2D-CEUS examination. One case was excluded due to loss in follow-up; thus 52 cases of adnexal tumors were included in the study.

*3.2.* Pathological and long-term follow-up results for the 52 adnexal masses are shown in Table 3: the mean size of the 52 cases was 3.3 ± 0.2 cm × 2.3 ± 0.1 cm × 3.0 ± 0.2 cm and, among them, 23 cases were diagnosed as AT-OCLH, three of which were confirmed by surgical pathology, while the other 20 cases were confirmed by long-term follow-up. There were 29 cases diagnosed as nonluteal hematoma, 28 of which were confirmed by surgical pathology and the other was confirmed by long-term follow-up.

*3.3. Features of AT-OCLH.* 2D gray scale results from 23 cases showed that masses manifested as mixed (messy and inhomogeneous hypoechogenicity) or nearly inhomogeneous solid echo, and CDFI showed blood flow signal in the periphery and interior of the mass, part of which led to an arterial blood flow signal spectrum, with a Resistant Index (RI) value between 0.28 and 0.55.

**Figure 2:**
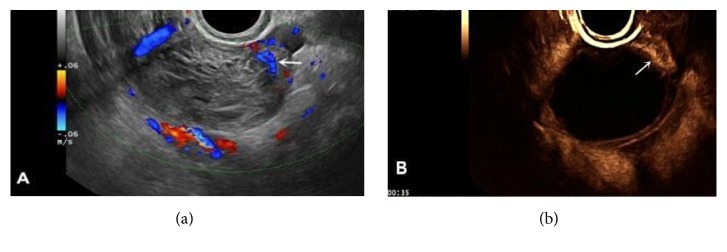
AT-OCLH (a) 2D gray-scale and CDFI examination: the mass showed inhomogeneous solid echogenicity. As the cyst wall was thick, blood flow on the cyst wall could be misdiagnosed as vessels within the tumor (arrow). (b) 2D-CEUS: annular perfusion with no contrast agent perfusion in the mass, indicating that the misdiagnosed vascular flow is big nourishing blood vessels (arrow), and the perfusion intensity of the cystic wall is slightly higher than the surrounding tissue.

The 2D-CEUS results showed that there was no contrast agent perfusion inside the mass, while the cyst wall demonstrated rapid, annular, and high intensity perfusion, and nourishing vessels were clearly observed on parts of the cyst wall. The perfusion pattern was type Ia and type IIa (4 and 19 cases, respectively); the perfusion intensity was mostly grade 3 (20 cases of grade 3 and three cases of grade 2); the cyst wall of mass was relatively thick, with M (*P*_25_, *P*_75_) of 4.3 mm (3.2, 5.0 mm) (Figure 2).

**Figure 3:**
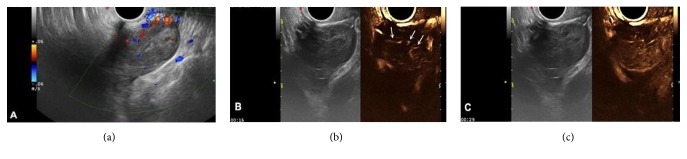
Stage Ia ovarian granulosa cell tumor. (a) 2D gray-scale and CDFI showed a mass with medium or low echo and scattered blood flow signal. (b) 2D-CEUS showed the first internal perfusion area at 16 s (arrow) and (c) full perfusion of the whole mass at 29 s with inhomogeneous perfusion.

*3.4. Characteristics of Masses Other than AT-OCLH*. (1) There were eight cases of malignant tumors, including six cases showing solid medium or hypoechogenic and two cases showing mixed echogenic foci by 2D gray scale examination. CDFI showed blood flow signal at the edge and inside of the mass, mostly leading to arterial blood flow signal spectrum, with an RI value of 0.36–0.56. The 2D-CEUS results showed that the interior of the solid mass had contrast agent perfusion, showing rapid inhomogeneous perfusion, or mixed mass with thick septa and papilla perfused with contrast agent. The perfusion patterns were classified as types III–IV (two cases of type IIIa, two cases of IIIb type, and four cases of type IV). The perfusion intensity was grade 3 (Figure 3).

**Figure 4:**
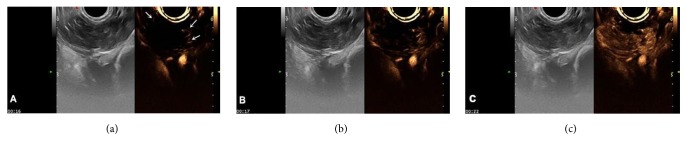
Theca fibroma 2D-CEUS: (a) At 16 s, the edge of the mass started to show perfusion (arrow). (b) Homogeneous perfusion from the outside to the inside of the mass was observed at 17 s. (c) Complete and homogeneous perfusion of the mass at 22 s.

**Figure 5:**
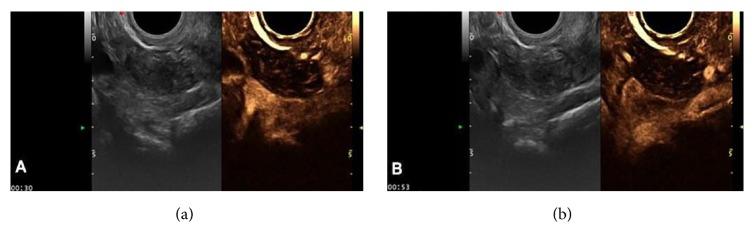
Intraligamentary myoma showing contrast agent perfusion within the mass. (a) Sparse reticular perfusion of the tumor at 30 s. (b) 2D-CEUS showed decreased mass perfusion intensity at 53 s.

**Figure 6:**
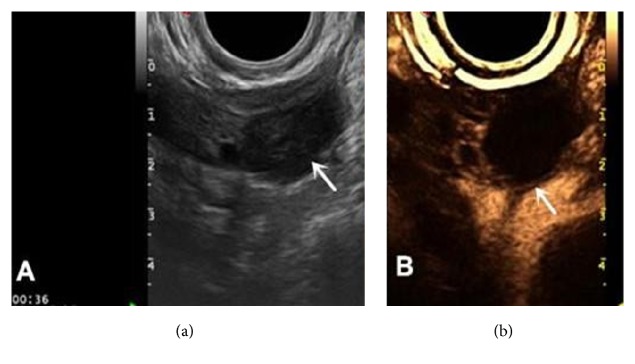
Chocolate cyst. (a) Image showing hypoechoic lesion (arrow). (b) 2D-CEUS showed no perfusion in the cyst with relatively thin wall (arrow), and the wall perfusion intensity was lower than the surrounding tissue.

**Figure 7:**
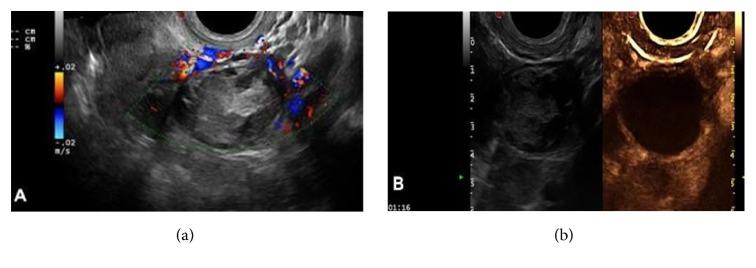
Mature cystic teratoma. (a) Image showing a hypoechoic lesion with strip blood flow signal on the cystic wall. (b) 2D-CEUS showing no perfusion in the cyst, and the perfusion intensity of the cystic wall was lower than the surrounding area.

(2) There were seven cases of theca fibroma, in which the 2D gray scale showed hypoechoic solid masses and no obvious posterior attenuation, and CDFI showed blood flow signal at the edge and within the masses, some of which led to arterial blood flow signal spectrum, with an RI value of 0.30–0.48. 2D-CEUS observations showed contrast agent perfusion of the whole parenchyma mass, mostly homogeneous perfusion from the margin to the center of mass (five cases), while two cases showed inhomogeneous perfusion, including one case with simultaneous perfusion of the central and peripheral tumor regions and one case showing inhomogeneous perfusion from the center to the peripheral of the mass. Perfusion patterns included types III–IV (five cases of IIIb and two cases of type IV). The perfusion intensity was 1–3 (two cases of grade 1, two cases of grade 2, and three cases of grade 3) (Figure 4).

(3) There were two cases of intraligamentary myoma, in which the 2D gray scale showed hypoechoic lesions with no obvious posterior attenuation. CDFI showed blood flow signal at the edge and within the mass, one of which led to an arterial blood flow signal spectrum, with an RI value of 0.39. 2D-CEUS showed contrast perfusion of the mass parenchyma, demonstrating sparse reticular perfusion. The perfusion pattern was classified as IIIb with grade 2 perfusion intensity (Figure 5).

(4) There were six cases of chocolate cyst, and 2D gray scale showed hypoechoic lesion, with two cases that led to artery spectrum with an RI of 0.44 for both. 2D-CEUS showed that there was no contrast agent perfusion within the mass and annular perfusion of the cyst wall. The perfusion pattern classification was type I; perfusion intensity was mostly grade 2 (five cases of grade 2 and one case of grade 3); cyst walls were relatively thinner, and M (*P*25,* P*75) was 2.1 mm (1.9, 2.5 mm) (Figure 6).

(5) There were six cases of mature cystic teratoma, in which 2D gray scale showed lesions with low or slightly high echogenicity, part of which led to artery spectrum, with RI values ranging between 0.24 and 0.49. 2D-CEUS showed no contrast agent perfusion within the mass, with slow, annular, low perfusion intensity of the cyst wall. Perfusion pattern classifications were mainly type Ia (four cases of type Ia, one case of type IIa, and one case of type IV); perfusion intensity was mostly grades 1–2 (three cases of grade 1, two cases of grade 2, and one case of grade 3). Cyst walls were relatively thin, with M (*P*_25_, *P*_75_) of 2.7 mm (2.3, 3.4 mm) (Figure 7).

*3.5. Differential Diagnosis of AT-OCLH with Ovarian Tumor or Intraligamentary Myoma*. Differential diagnoses were made based on contrast-enhanced perfusion and perfusion pattern classification. For perfusion characteristics, AT-OCLH showed annular perfusion, no contrast agent perfusion within the solid mass, while ovarian tumors and intraligamentary myomas demonstrated contrast agent perfusion of the solid mass. For perfusion pattern types, AT-OCLH was types Ia and IIa, with one case misdiagnosed as type III. Ovarian neoplasms and intraligamentary myomas were type IIIB and type IV, with statistically significant differences in perfusion patterns between the two,* P *< 0.05 (Table 4).

Differential diagnosis of AT-OCLH and malignant tumors: In this study, 18 of the 23 cases of AT-OCLH were determined with RI values by CDFI ranging between 0.28 and 0.55; six of eight cases of malignant tumors demonstrated RI values ranging between 0.36 and 0.56. There was no significant difference in the distribution of RI values between the two groups. These results showed that the RI value as determined by CDFI could not be used to distinguish AT-OCLH and malignant tumors (Table 5). However, these two can be distinguished by the perfusion characteristics and perfusion patterns shown by 2D-CEUS (Table 4).

*3.6.* Differential diagnosis of AT-OCLH and nonluteal cystic lesions (chocolate cyst and mature cystic teratoma): for perfusion features, all three types demonstrated annular perfusion. However, the typical features of AT-OCLH also included the following: (1) most of the AT-OCLH demonstrated nourishing vessels from the ovarian medulla; (2) AT in the AT-OCLH group was significantly earlier than the nonluteal cystic lesion group (*P *< 0.05); but there was no significant difference in TTP and PEAK (Table 6); (3) in terms of perfusion intensity, cyst wall perfusion intensity of the AT-OCLH group was stronger than the nonluteal cystic lesion group; the AT-OCLH group mainly showed grade 3 intensity, while the nonluteal cystic lesion group were mostly grades 1–2, which was a statistically significant difference (*P *< 0.05, Table 7); (4) in terms of wall thickness, walls of the AT-OCLH group were thicker, while walls of the chocolate cyst and mature cystic teratoma groups were statistically significantly thinner (*P *< 0.05, Table 8). Finally, the value of 2D-CEUS in diagnosing AT-OCLH is shown in Table 9.

*3.7.* The 2D-CEUS diagnostic scoring system for AT-OCLH (Table 10): A scoring system was developed based on six indicators according to the 2D-CEUS features of AT-OCLH, including the perfusion characteristics, perfusion pattern type, perfusion intensity, presence of perfusion vessels, AT, and wall thickness. The imaging scores of 52 masses are shown in Table 11.

**Figure 8:**
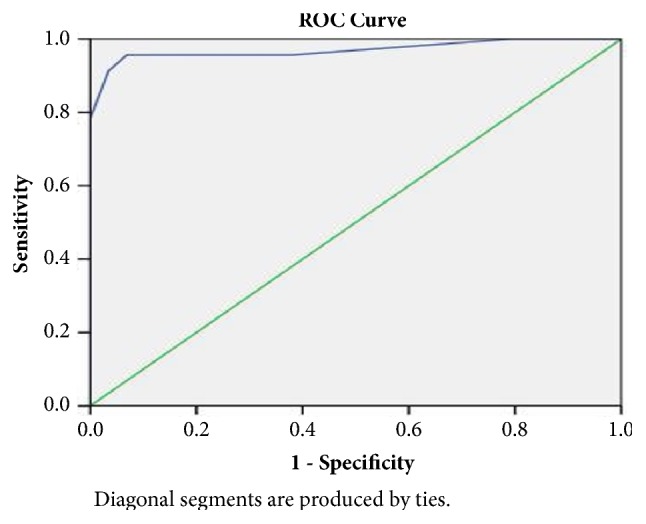
The ROC curve of the 2D-CEUS scoring system for AT-OCLH.

*3.8.* Receiver operating characteristic (ROC) curve analysis: The ROC curve of AT-OCLH contrast imaging system score was plotted as shown in Figure 8, and the largest area under the curve was 0.97, demonstrating high diagnostic accuracy for this scoring system in AT-OCLH. When 17.5 was set as the threshold, the diagnostic sensitivity was 91.3%, with a misdiagnosis rate of 3.4%. Therefore, >17.5 is likely a reasonable threshold for diagnosis of AT-OCLH.

## 4. Discussion

CEUS utilizes harmonic and pulse inversion techniques, with its frequency setting, and the probe can only accept nonlinear echo of microbubbles with specific enhancement, while filtering out the fundamental and intrinsic echo, which can effectively enhance the two-dimensional ultrasound imaging and flow Doppler signal of a parenchymatous organ, clearly showing tissue perfusion [16, 17]. Based on the characteristics of CEUS, we can perform a further 2D-CEUS examination of the solid echo lesion, which are difficult to distinguish by 2D gray scale or CDFI examination.

First, 2D-CEUS can be used to distinguish whether there are active tissues within the solid mass. The contrast agent perfusion within the solid tumors and intraligamentary myoma demonstrated perfusion patterns of type IIIb–IV. AT-OCLH, chocolate cyst, and mature cystic teratoma showed no contrast agent perfusion inside, with perfusion patterns of types I–II a. These two kinds of masses can be clearly distinguished based on perfusion characteristics and perfusion patterns. Among the 52 solid or mixed mass cases (messy and inhomogeneous hypoechogenic masses), 17 cases were not cystic lesions based on the perfusion of internal active tissue components shown by 2D-CEUS, including eight cases of malignant tumors, seven cases of theca fibroma and two cases of intraligamentary myoma. In total, 33 cases were determined as cystic lesions without active tissue perfusion inside, including 22 cases of AT-OCLH, six cases of chocolate cyst, and five cases of mature cystic teratoma. Two cases were misdiagnosed as solid masses with active tissue perfusion. The diagnostic accuracy of solid tissue with or without active tissue inside by 2D-CEUS was approximately 96.2% among the 52 cases.

**Figure 9:**
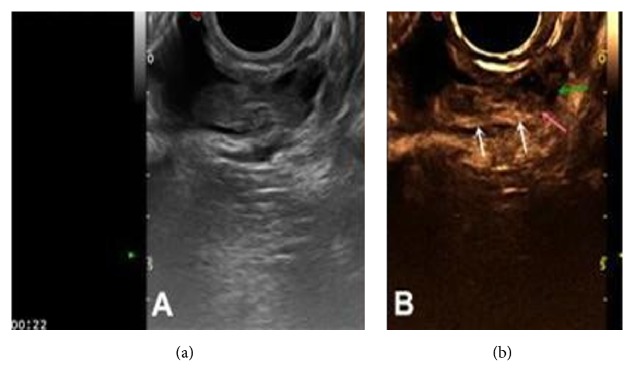
AT-OCLH. (a) Mixed echogenic foci; (b) ovarian tissue in the background of effusion (green arrow points to the follicle, pink arrow points to the ovarian medulla) and convex corpus luteum (white arrow points to the nourishing blood vessels originating from the ovarian medulla and cyst wall).

Second, 2D-CEUS was utilized to further diagnose AT-OCLH among the cystic lesions. In this study, cystic lesions were mainly AT-OCLH, chocolate cyst, and mature cystic teratoma. Although all three of them demonstrated annular perfusion without internal perfusion by 2D-CEUS, AT-OCLH had some differences compared with the other two: (1) most AT-OCLH demonstrated nourishing vessels originating from the ovarian medulla (see Figures 2 and 9), which is not observed in chocolate cysts or mature cystic teratomas (see Figures 6 and 7). (2) Due to the large amount of angiogenesis during luteal hematoma formation, which convey high speed and low resistance vascular hemodynamics, the blood flow speed of AT-OCLH was quicker than the other two, demonstrating significantly earlier AT values (Table 6). (3) Due to the large amount of angiogenesis during luteal hematoma, the cyst wall of AT-OCLH was also rich in blood supply, demonstrating a significantly stronger perfusion intensity of the cystic wall compared with the other two groups (Table 7). The cystic wall perfusion of mature cystic teratomas was the weakest, which is a characteristic of mature cystic teratomas. (4) The cystic wall of AT-OCLH was significantly thicker than the other two (Table 8). Based on these four points, AT-OCLH can be distinguished among cystic lesions with annular perfusion. There were a total of 35 cases of cystic lesions in this study. Excluding the two cases misdiagnosed as solid tumors, one case of chocolate cyst was misdiagnosed as AT-OCLH, and 32 cases of cystic lesions were correctly diagnosed, yielding a positive detection rate of 91.4%.

Even after the aforementioned two steps, we still could not determine whether some of the cystic lesions were AT-OCLH that required observation over long-term follow up. One case of cysts showed nourishing vessel on the cystic wall with relatively strong perfusion intensity and a 2D-CEUS score of 18 points, leading to a diagnosis of AT-OCLH. However, this cyst did not disappear after 6 months of follow-up, and was significantly reduced after clinical treatment for chocolate cyst, leading to a chocolate cyst diagnosis.

**Figure 10:**
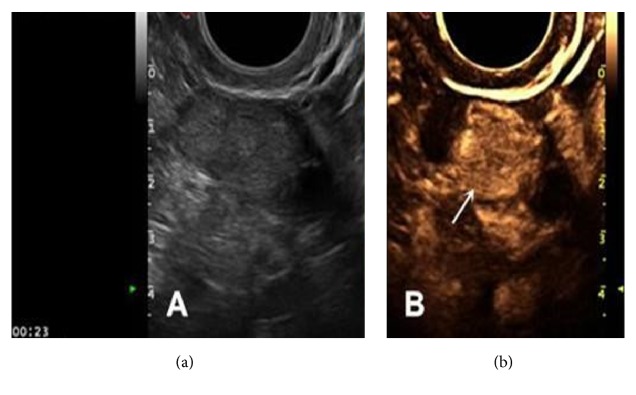
A mature cystic teratoma. (a) 2D gray scale examination demonstrated hypoechogenic foci. (b) Hypoechoic foci with active tissue perfusion (arrow).

In this study, two cases of cystic lesions were misdiagnosed as solid masses with active tissue within. Among them one case of AT-OCLH was misdiagnosed as a malignant tumor (Figure 9). This mass showed as mixed echogenic foci in the 2D gray scale examination, while 2D-CEUS showed that the solid mass was inhomogeneously perfused by contrast agent and was therefore diagnosed as a malignant tumor. After it was confirmed as luteal hematoma through postoperative pathological results, we carefully played-back the dynamic image and found that in the background of effusion with no echogenicity we mistook the thick convex ovarian corpus luteum as a mixed echogenic lesion with solid mass perfusion. Additionally, this corpus luteum with thick walls demonstrated typical perfusion characteristics of corpus luteum hematoma, including annular perfusion, nourishing vessels originating from the ovarian medulla, thick cystic walls, and a high perfusion intensity (grade 3, similar to the perfusion intensity of iliac vessels). Another mature cystic teratoma was misdiagnosed as a malignant tumor (Figure 10). This hypoechogenic foci showed inhomogeneous internal active tissue perfusion, and therefore was diagnosed as a malignant tumor. Postoperative pathological results suggested that it was a mature cystic teratoma of mainly thyroid tissue. The presence of solid components in the thyroid tissue with contrast agent perfusion, which was a rare case, led to the misdiagnosis.

The application of 2D-CEUS for the differential diagnosis of AT-OCLH and malignant ovarian tumor demonstrates obvious advantages in comparison to the conventional 2D gray scale and CDFI examination [18, 19]. In the 2D gray scale examination, both AT-OCLH and malignant ovarian tumors showed solid echo foci, while CDFI showed various amounts of blood flow signal inside and around the lesion, which are easily confused (panel A of Figures 2 and 3). Kurjak et al. [20] have reported that the RI value determined by CDFI can be used to diagnose the tumor malignancy, with RI ≤ 0.4 as the diagnostic threshold for malignant ovarian tumors. However, for AT-OCLH, due to the large amount of angiogenesis on cystic walls, its blood flow shows high speed and low resistance [21], the blood RI value of which was low. Tumor blood vessels have a paucity of muscular media compared with normal vessels and are more distensible. This combined with arteriovenous shunts seen in the tumor vascular network results in low impedance flow [21]. Therefore, it is difficult to distinguish malignant tumors and AT-OCLH based solely on RI values (Table 5). Using 2D-CEUS, we can clearly distinguish between malignant tumors and cystic lesions based on the small blood vessels shown by contrast agent enhancement.

In summary, through 2D-CEUS, the diagnosis of AT-OCLH using the six indicators, perfusion characteristics, perfusion pattern type, perfusion intensity, presence of perfusion vessels, AT, and wall thickness, demonstrated a sensitivity of 95.7%, a specificity of 96.6%, a positive likelihood ratio of 27.74, a negative likelihood ratio of 0.05, a positive predictive value of 95.7%, a negative predictive value of 96.6%, and a coincidence rate of 96.2 (Table 9).

The area under the ROC curve for the AT-OCLH contrast-enhanced ultrasound scoring system based on these six indicators was 0.97, indicating a relatively high diagnostic accuracy. With a threshold of 17.5 points, the diagnostic sensitivity was 91.3% and the misdiagnosis rate was 3.4%, demonstrating a satisfactory diagnostic efficacy for AT-OCLH.

## 5. Conclusion

The 2D-CEUS is an effective method for diagnosing AT-OCLH and has a higher practical value compared with the conventional 2D gray scale and CDFI examinations. The 2D-CEUS scoring system also demonstrated a high diagnostic accuracy, which makes it worth pursuing in clinical settings.

## Figures and Tables

**Table 1:** Perfusion patterns in adnexal masses on 2D-CEUS.

Pattern		characteristics
Type I	Cyst wall perfusion, thin wall (<3 mm)	A: no perfusion in cyst B: fine septa (≤1 mm) perfusion visible within the cyst
Type II	Cyst wall perfusion, thin wall (≥3 mm)	A: no perfusion in cyst B: perfusion of papilla on inner wall of the cyst (< 3 mm) and/or incomplete/complete septa perfusion, with uneven thickness (1–3 mm)
Type III	Perfusion within the cyst	A: perfusion of papilla on inner wall of the cyst (≥3 mm) and/or incomplete/complete septa perfusion, with uneven thickness, which can be ≥3 mm B: perfusion from periphery to the center of the tumor, or homogeneous perfusion of the mass, or sparse network perfusion of blood vessel
Type IV	Perfusion within the cys	Inhomogeneous perfusion from one or more points in the mass, with or without perfusion defect area within the mass

**Table 2:** Grading perfusion intensity by contrast-enhanced ultrasound.

Grade 1	Slight enhancement	Perfusion within the mass but significantly lower enhancement compared with surrounding tissues
Grade2	Moderate enhancement	Perfusion within the mass with similar enhancement compared with surrounding tissues
Grade 3	High enhancement	Perfusion within the mass and significantly higher enhancement compared with surrounding tissues, similar to or higher than the perfusion intensity of iliac vessels

**Table 3:** Pathological and long-term follow-up results for the 52 patients with adnexal masses suspected to be AT-OCLH.

Group	Cases (N)
AT-OCLH	23
Non-luteal hematoma group	29
Non-luteal cyst group	12
Chocolate cyst	6
Mature cystic teratoma	6
Non-luteal solid mass group	17
Theca fibroma (atypical*∗*)	7
Intraligamentary myoma	2
Malignant tumor	8
Stage Ia ovarian granulosa cell tumor	2
Stage Ia ovarian adenocarcinoma	1
Stage Ia ovarian Sertoli-Leydig cell tumor	1
Stage IIc tubal serous adenocarcinoma	1
Ovarian serous carcinoma T3bNxM1	1
Borderline ovarian tumor	1
Pelvic metastases (malignant)	1
Full	52

**Table 4:** Comparing 2D-CEUS perfusion patterns in AT-OCLH and nonluteal solid masses.

Group	Cases (N)	Classification of perfusion pattern			
		Type I	Type II	Type III	Type IV
AT-OCLH	23	4	18	1	0
Non-luteal solid masse	17	0	0	11	6
U value			5.500		
*P*			<0.001		

*Note.* Mann–Whitney U test, P < 0.05.

**Table 5:** Comparing RI values in AT-OCLH and malignant tumors group.

Group	Cases (N)	RI		
		≤0.4	0.4–0.5	≥0.5
AT-OCLH	18	6	9	3
Malignant tumor	6	2	3	1
U value			54.000	
*P*			1.000	

**Table 6:** Comparing TIC parameters M (*P*_25_, *P*_75_) between AT-OCLH and nonluteal cystic lesions.

Group	Cases (N)	AT (s)	TTP (s)	PEAK (%)
AT-OCLH	23	12.0 (11.0,15.3)	24.1 (20.7,26.5)	39.7 (28.2,56.0)
Non-luteum	12	16.5 (11.7,20.0)	24.3 (19.0,35.6)	26.2 (21.8,44.4)

U value		72	104	84
*P*		0.031*＊*	0.424	0.123

*Note.* Mann-Whitney U test, *P *< 0.05. AT: arrival time of contrast agent; PEAK: peak intensity; TTP: Time-to-Peak.

**Table 7:** Comparing the perfusion intensity between AT-OCLH and nonluteal cystic lesions.

Group (N)	Cases	Perfusion intensity		
		Grade 1	Grade 2	Grade 3
AT-OCLH	23	0	3	20
Non-luteum cystic lesion group	12	3	7	2
U value			36.5	
*P*		< 0.001		

*Note.* Mann–Whitney U test, *P *< 0.05.

**Table 8:** Comparing cystic wall thickness M *(P*_25_, *P*_75_) between AT-OCLH and non-luteal cystic lesions.

Group	Cases (N)	Cystic wall thickness (mm)	U	P value
AT-OCLH	12	4.3 (3.2 5.0)		
Chocolate cyst	6	2.1 (1.9 2.5)	10.500	0.001
Mature cystic teratoma	6	2.7 (2.3 3.4)	22.500	0.009

*Note.* Mann–Whitney U test, *P *< 0.05.

**Table 9:** Value of 2D gray scale + CDFI and 2D-CEUS in diagnosing AT-OCLH.

Method	N (Case)	TP	FP	TN	FN	Sen (%)	Spe (%)	LR^+^	LR^−^	PV^+^ (%)	PV^−^ (%)	Consist ency (%)
2D-CEUS	52	22	1	28	1	95.7	96.6	27.74	0.05	95.7	96.6	96.2
2D ray-scale + CDFI	52	10	14	15	13	43.5	51.7	0.90	1.09	41.6	53.5	48.1

*Note.* TP: true positive; FP: false positive; TN: true negative; FN: false negative; Sen: sensitivity; Spe: specificity; LR+: positive likelihood ratio; LR-: negative likelihood ratio; PV+: positive predictive value; PV-: negative predictive value.

**Table 10:** 2D-CEUS scoring system for the diagnosis of AT-OCLH.

Score (point)	Perfusion characteristics	Perfusion pattern	Perfusion intensity	Nourishing vessels	AT^*^(s)	Cystic wall thickness (mm)
1	Solid	Type I	Grade 1	without	≥20	NO
	perfusion					
2	/	Type II	Grade 2	/	17–19	≤3
3	/	Type III	Grade 3	/	15–16	3.1–3.9
4	Annular	Type IV	/	with	≤14	≥4
	perfusion					

*Note.* ^*∗*^AT: arrival time of contrast agent in the lesions; see Table 1 for perfusion pattern classification; see Table 2 for perfusion intensity classification.

**Table 11:** 2D-CEUS scores of the 52 adnexal masses.

Group	Cases (n)	Score M (*P*_25_, *P*_75_)
AT-OCLH	23	21 (19, 23)
Malignant tumor	8	11 (11, 11)
Theca fibroma	7	10 (9, 11)
Intraligamentary myoma	2	9.5 (8, 11)
Chocolate cyst	6	16 (14.8, 17.3)
Mature cystic teratoma	6	14 (12.5, 14)

Full	52	

## Data Availability

The data used to support the findings of this study are available from the corresponding author upon request.
